# Dissociated Emergent-Response System and Fine-Processing System in Human Neural Network and a Heuristic Neural Architecture for Autonomous Humanoid Robots

**DOI:** 10.1155/2010/314932

**Published:** 2011-02-10

**Authors:** Xiaodan Yan

**Affiliations:** ^1^Cognitive Science Department, Rensselaer Polytechnic Institute, Troy, NY 12180, USA; ^2^Langone Medical Center, New York University, New York, NY 10016, USA

## Abstract

The current study investigated the functional connectivity of the primary sensory system with resting state fMRI and applied such knowledge into the design of the neural architecture of autonomous humanoid robots. Correlation and Granger causality analyses were utilized to reveal the functional connectivity patterns. Dissociation was within the primary sensory system, in that the olfactory cortex and the somatosensory cortex were strongly connected to the amygdala whereas the visual cortex and the auditory cortex were strongly connected with the frontal cortex. The posterior cingulate cortex (PCC) and the anterior cingulate cortex (ACC) were found to maintain constant communication with the primary sensory system, the frontal cortex, and the amygdala. Such neural architecture inspired the design of dissociated emergent-response system and fine-processing system in autonomous humanoid robots, with separate processing units and another consolidation center to coordinate the two systems. Such design can help autonomous robots to detect and respond quickly to danger, so as to maintain their sustainability and independence.

## 1. Introduction

In the research community on human level intelligence [1, 2], there has been increasing investigation on autonomous agents [3] and humanoid robots [4, 5], for which independent survival is essential. Previously, research efforts have been focused on imitating human cognition and behaviors (for review see [6]), for example, motion, perception, reasoning, and even emotion and social interaction [6–9], but no sufficient attention has been paid upon their sustainability, for example, monitoring and avoiding danger, acknowledging physical harm and threatening, and so forth. The current study hopes to apply the knowledge from the neural network of the human brain into the design of the neural architecture of humanoid robots. To be autonomous, the robot needs to maintain constant monitoring of its outside environment and inside status, which is similar to the function of the primary sensory system of the human brain; it is also better to have an independent processing unit so as to respond quickly in face of danger, which is similar to the role of the amygdala in the human brain. It is also necessary to have an executive center to consolidate the possible conflict between the need for survival-based quick response and the need for thorough computation, similar to the cognitive control role of the anterior cingulate cortex (ACC). Therefore, we hope to gain some insight from the neural architecture of the human brain to help such design. 

Functional MRI (fMRI) has allowed us to reveal *in vivo* the architecture of the neural network in human brain via analysis on the functional connectivity between different brain regions [10, 11]. It has been confirmed that the spontaneous low-frequency oscillation in the frequency band of 0.01 ~ 0.08 in the BOLD fMRI signal can reflect the temporal synchronization among functionally related brain regions [12–15]. Such functional connectivity in the resting state has attracted a lot of attention in the neuroscience community as a potentially reliable, task-independent assay for global circuit function [16–18]. Therefore, the current study investigated the functional connectivity with resting state fMRI. 

Regions of interest (ROIs) were selected among the primary sensory regions, the amygdala, the prefrontal cortex, the posterior cingulate cortex (PCC), and the ACC. The amygdala is a well-known processing center for fear and relevant response [19, 20], whereas the prefrontal cortex is important for reasoning and working memory [21, 22]. The PCC is considered as essential for evaluative processing such as monitoring the outside and inside world [23, 24]. The ACC has been found to be an interface for cognitive control, conflict monitoring, and so forth, [25, 26]. Therefore, the current study aims to investigate the functional connectivity among the primary sensory system and their communication with the amygdala, the PCC, and the ACC, under the hypothesis that there are two dissociated systems in the neural network of human brain: one is for emergent-response processing to detect and respond to danger via the amygdala, and the other is slow thorough and detailed processing via the frontal cortex. There might be corresponding dissociation among the primary sensory system in terms of their connectivity with the two processing centers. The ACC and PCC should maintain functional connectivity with these regions considering their role in information integration and cognitive control. Correlation analysis and Granger causality analysis were applied to assess the functional connectivity. Correlation analysis is unidirectional; it reflects the temporal synchronization between the regions under investigation, whereas Granger causality analysis can reflect the direction of information flow. Application of both analyses allows for overview of the whole connectivity patterns as well as getting a glimpse of the information flow direction. 

## 2. Methods

A seven-minute resting state fMRI data were acquired from 29 subjects on a GE 3.0 T Signa Excite Gemse MRI system (GE Medical, Milwaukee, WI, USA) with an EPI sequence (TR/TE = 2000/30 ms, FOV = 24 × 24 cm^2^, flip angle = 90°, matrix 64 × 64). Corresponding structural MRI was also acquired on the same scanner. Totally 200 volumes of 28 contiguous axial slices at 5 mm thickness (without gaps) covering the whole brain were acquired from each subject (subjects were instructed to remain awake with their eyes closed). The original voxel size was 3.75 mm × 3.75 mm × 5 mm. The following preprocessing was conducted on the data of each subject with AFNI [27]: discarding the first 10 volumes for scanner calibration, slice timing, motion correction, removal of linear drift, bandpass filtering (0.01–0.08 Hz), calculating the temporal signal change ratio, smoothing with a Gaussian filter of 4 mm full width at half maximum (FWHM), masking off the nonbrain voxels, and normalizing to the Talairach and Tournoux space [28], and the images were resampled into a cubic voxel size of 3 mm × 3 mm × 3 mm. None of the subjects' head motion exceeded 1 voxel, so no subject was excluded. The data was obtained from the 1000-connectome open data source (http://www.nitrc.org/).

The ROIs were outlined on the high-resolution structural MRI of each subject: the left and the right sides of the primary visual cortex (the lingual cortex), the primary auditory cortex (BA41), the primary motor cortex (the precentral cortex), the primary somatosensory cortex (the postcentral cortex), the olfactory cortex, the prefrontal cortex, the ACC, and the PCC with reference to the template provided in AFNI. The average time series were extracted from each ROI on each subject. The Pearson correlation coefficients (*r*) between the time series of each pair of ROIs in each individual subject were calculated:

(1) $$r=\frac{\sum_{i=1}^{n}\left(X_{i}-\bar{X}\right)\left(Y_{i}-\bar{Y}\right)}{\sqrt{\sum_{i=1}^{n}{\left(X_{i}-\bar{X}\right)}^{2}}\sqrt{\sum_{i=1}^{n}{\left(Y_{i}-\bar{Y}\right)}^{2}}}.$$

**Figure 1:**
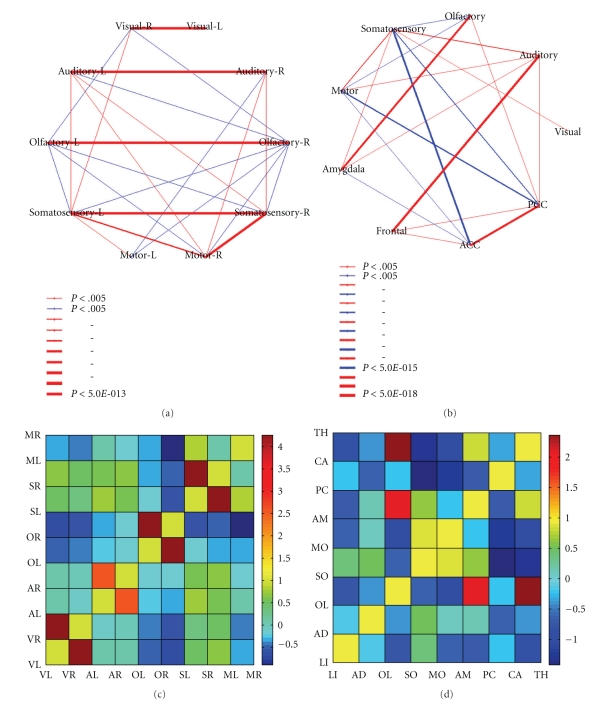
Illustration of the connections in the network. (a) The significant connections within the primary sensory network. (b) The significant connections between the primary sensory network and other regions. In (a) and (b), the line thickness indicates the significance level as labeled on the right of each figure. (c) The *Z*-matrix of the primary sensory network, reflecting the average correlation coefficient standardized by cross-subject variation. The labels were abbreviated with the first letter of each ROI used in Figure 1(a), with L, R indicating the left or right sides. (d) The *Z*-matrix corresponding to Figure 1(b), obtained in the same way as Figure 1(c). The labels were abbreviated with the first two letters of each ROI in Figure 1(b).

For group analysis, we further transformed the individual Pearson correlation coefficients into *Z* scores with Fisher *r*-*z* transform [29]

(2) $$z=\frac{1}{2}\ln\;\frac{1+r}{1-r}=\arctan\;h\left(r\right)$$

and further calculated the average and standard deviation of the *Z* scores, based upon which we obtained final *Z* scores (*Z*-matrix) reflecting the average correlations normalized by the deviation among all subjects (average divided by standard deviation), as plotted in Figures 1(c) and 1(d). Since the standard deviation was 0 for self-correlation, we set the diagonal elements in the *Z* matrix to 1 by hand. The significant *z*-scores between each pair of ROIs were plotted upon the graphs in Figures 1(a) and 1(b), with the line thickness indicating different levels of significance. Bonferroni correction was used to correct for multiple comparison in determining the significance levels, and the threshold for significance was *P* < .005.

Granger causality analysis is an approach for determining whether one time series is effective in predicting another. Unlike regression analysis which reflects correlations only, Granger causality analysis allows for interpretation on the causality of two time series. The mathematical basis for Granger causality analysis was well explained in previous studies [30, 31]. The basic principle is to test whether knowing the past of *X* can help predict *Y* better than knowing the past of *Y* alone; if so, a statistical interpretation can be made that *X* “Granger causes” *Y*. The Granger causality of two variables can be implemented based on two linear regression functions. A restricted model for prediction of *Y* by its own past can be expressed as follows:

(3) $$Y\left(t\right)=\alpha_{1}Y\left(t-m\right)+\varepsilon_{1}\left(t\right),$$

where *m* is the time lag considered for estimation, and the unrestricted model for prediction of *Y* by the past of *X* can be expressed as follows:

(4) $$Y\left(t\right)=\alpha_{2}Y\left(t-m\right)+\beta X\left(t-m\right)+\varepsilon_{2}\left(t\right).$$

An *F* test can be conducted to test whether the residual of the unrestricted model (4) is significantly reduced than that of the restricted model (3):

(5) $$F=\frac{\left({\text{RSS}}_{r}-{\text{RSS}}_{ur}\right)/m}{{\text{RSS}}_{ur}/\left(T-2m-1\right)},$$

where RSS_*r*_ is the sum of squares for the restricted model and RSS_*ur*_ is the sum of squares for the unrestricted model. *T* is the total number of observations. If the *F* statistic is significant, an interpretation can be made that *X* “Granger causes” *Y*. 

**Figure 2:**
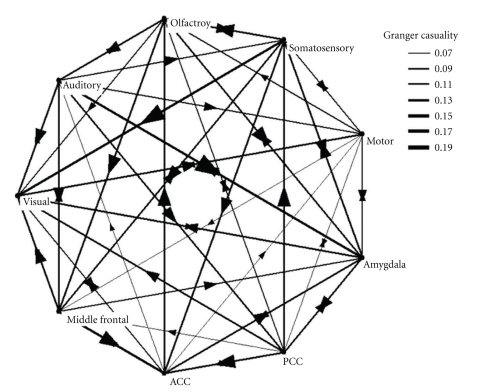
The information flow in the neural network under investigation, obtained from Granger causality analysis.

The mathematical basis for multivariate estimation is much more complicated. The current study utilized the implementation via multiple vector autoregressive (MVAR) modeling with the assistance of the Granger Causal Connectivity Analysis Matlab Toolbox [32] and the BSMART Toolbox [33]. For the original time series from all the ROIs in each subject, detrending and demeaning were conducted as preprocessing steps, to subtract the best-fitting line and to remove the temporal mean. Covariance stationarity was tested on the time series matrix; as an effort to restore the covariance stationarity, we used successive time window of 60 seconds (30 time points); each segment was covariance stationary, so we conducted Granger causality analysis on each; since multiple pairs of ROIs were involved in the granger causality analysis, Bonferroni correction was used to correct for multiple comparison in determining the significance levels, and the threshold for significance was set at *P* < .001. The causal influence estimation from the Granger causality analysis was averaged in the group and plotted with the Pajek network software (http://vlado.fmf.uni-lj.si/pub/networks/pajek/), giving rise to Figure 2. 

## 3. Results

### 3.1. Functional Connectivity within the Primary Sensory System

During waking rest, significant correlations between the left and the right correspondence of a functional brain region robustly appeared significantly among all subjects [12, 34]. Dissociation was found in the connectivity patterns within the primary sensory cortex; for example, the olfactory cortex maintains a negative correlation with all other primary sensory cortices, the visual cortex and the auditory cortex were positively correlated with the somatosensory cortex, and the auditory cortex was positively correlated with the motor cortex (see Figure 1(a)). 

### 3.2. Functional Connectivity between the Primary Sensory System and Other Regions

Dissociation was also found among the correlations of the ROIs in the primary sensory system and the other regions. The ACC was found to be negatively correlated with the visual cortex, the motor cortex, and the somatosensory cortex but positively correlated with the frontal cortex and the PCC. The PCC was negatively correlated with the motor cortex and the somatosensory cortex but positively correlated with the olfactory cortex, and the auditory cortex. The amygdala was positively correlated with the olfactory cortex, the auditory cortex, and the somatosensory cortex. The frontal cortex was positively correlated with the auditory cortex, but negatively correlated with the visual cortex. The frontal cortex was positively correlated with the PCC and the ACC, whereas the amygdala was negatively correlated with the PCC and the ACC (Figure 1(b)). 

### 3.3. The Information Flow in the Neural Network

The information flow in the neural network under investigation was revealed through the Granger causality analysis (Figure 2), in which the following causality pattern was demonstrated: the amygdala maintains bidirectional communication with the olfactory, the auditory, the somatosensory, the visual, and the motor cortices. The frontal cortex receives information from the olfactory cortex and the motor cortex and maintains bidirectional communication with the visual cortex, the auditory cortex, and the somatosensory cortex. The PCC receives information from the olfactory cortex and the motor cortex and sends information to the visual cortex and the somatosensory cortex and maintains bidirectional communication with the amygdala. The ACC receives information from the frontal cortex and the PCC and maintains bidirectional communication with the amygdala, and sends information to the auditory, olfactory, and the somatosensory cortex. Such results support the hypothesized information flow in the primary sensory system and related regions; for example, the amygdala and the frontal cortex receive information from the primary sensory regions and send information to the PCC and the ACC. The visual cortex also seems to play an important role in information integration in that it receives information from other primary sensory regions as well as the frontal cortex, the amygdala, the ACC, and the PCC. 

### 3.4. A Heuristic Model for Humanoid Robot Design

**Figure 3:**
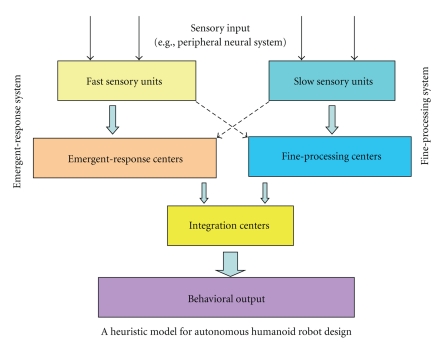
A heuristic model for autonomous humanoid robot design, with two dissociative systems: the emergent-response system and a fine-processing system. Regions of the human cerebral cortex were used as examples for sensory units and the processing centers.

The connectivity and causality patterns found above are inspiring for the architecture of an autonomous humanoid robot. Dissociation between an emergent-response system and a fine-processing system is proposed. The emergent-response system is consisted of specialized sensory units (such as the olfactory system in human) and an emergent-response center (similar to the role of the amygdala in human). The fine-processing system consists of a fine-processing center (similar to the frontal cortex of human brain) and corresponding sensory inputs (such as the visual system). There is constant communication between them. There are also integration centers that integrate the information from the two systems and consolidate possible confliction between them (Figure 3). 

## 4. Discussion

The current study explored the functional connectivity of the primary sensory system and their connectivity with the amygdala, the frontal cortex, the PCC, and the ACC. Dissociation was found in the connectivity patterns. The amygdala was found to be strongly connected with the olfactory cortex and the somatosensory cortex, compared to the frontal cortex which was strongly connected to the visual cortex and auditory cortex. The PCC and the ACC were found to play important roles in bridging the frontal cortex and the amygdala as well as the primary sensory system. 

The dissociation indicates the existence of two dissociated primary sensory systems. There may exist two systems in the neural network of the human brain; one is an emergent-response system, which monitors the outside world, detects danger, and responds very quickly without complicated computation, and the other is a fine-processing system, which processes sensory input information in fine detail and responds based on slow and thorough computation. The olfactory cortex and the somatosensory cortex might serve for the emergent-response system by maintaining bidirectional communication with the amygdala whereas the visual and the auditory cortices might belong to the fine-processing system by maintaining strong connectivity with the frontal cortex. Such dissociation is possible to have evolutionary necessity. Under circumstances when survival is threatened, emergent-response should be taken (such as flee) and fine-processing would be unnecessary. Olfaction and somatosense are very important for animals to avert rotten or poisonous food and physical harm; thus, it is reasonable for them to have a strong connection with the amygdala. On the other hand, the visual cortex has been found to be the major information input for human cognition [35, 36], and many functionally selective regions have been found at the cortex associated with visual cognition (e.g., the fusiform cortex) [37] such as regions selective for face [38], objects [39], and words [40]. The auditory cortex is another important input for human cognition [41]. Therefore, the visual and the auditory cortices are representative sensory input for the fine-processing system. Recent progress in vision science further proposed that the visual and associated cortices play such an important role in various kinds of cognition; they might also serve as cognitive hubs besides the frontal cortex [42], and the connectivity patterns in the current study indeed revealed that the primary visual cortex receives information from many brain regions, yet to be conservative, we still put the frontal cortex as an example of the fast processing center in Figure 3. 

The dissociation between the emergent-response system and the fine-processing system, with the amygdala and frontal cortex serving as processing centers for the above-mentioned representative primary sensory regions, is inspiring for designing similar dissociated systems in autonomous humanoid robots. Current robots usually rely on one single sensory input, usually vision; this is not sufficient for responding quickly to danger from the outside world. Integration of multiple sensory input, with some being primarily for detecting danger, could be crucial for the survival of autonomous robots. For example, an auditory unit and a tactile sensory unit can be designed to sense the approaching of other objects when they are out of the visual field (e.g., in the situation when the visual field is blocked, or when the object is too far to be detected by vision). Accompanying processing units should also be designed to enable the robots to react quickly when suspected danger is close. 

Besides designing independent fast-processing units to detect and respond to danger, it is also necessary to include processing units to integrate the multisensory input information and to consolidate the competition between the fast processing units and fine-processing units, to ensure the efficiency of the neural architecture of autonomous robots. In the human brain, the PCC and the ACC play such a role in the integration of sensory input and resolving the confliction between the two systems. The PCC has been found to be a region that is constantly active during the resting state, and it is also a representative region of the default mode network, a network that has higher activity during resting state than during cognitive tasks, and was hypothesized to be monitoring the outside world and getting ready to respond [16]. The PCC has been found to play an important role in evaluating the current status of the outside and inside world [23] as well as processing of emotional information [43]. In the current study, the PCC was found to maintain connectivity with the primary sensory regions, the amygdale, and the frontal cortex, which supports its proposed role in information integration. On the other hand, ACC has been found to play an important role in cognitive control [26, 44] and mediating attention conflict [45] as well as being an important interface between emotion and cognition [46]. In the current study, the ACC was found to receive information from the primary sensory regions, as well as the PCC and the frontal cortex, and maintain bidirectional communication with the amygdala, which is consistent with its well-known role in cognitive and attention control. Similar independent processing units serving such purposes for autonomous robots are also necessary to be included in their neural architecture (Figure 3). 

The functional connectivity analysis in resting state fMRI allows neuroscientists to study the complex neural network of human brain in large scale [47–49], making it possible to reveal the architecture of the neural network in human brain. Such knowledge can be inspiring for architecture design in artificial intelligence, yet application of such findings in artificial intelligence is lagged behind. The current study revealed the dissociation of an emergent-response system and a fine-processing system in the human brain neural network, with dissociated processing units and a unit for consolidating the confliction between the two systems. Such dissociation is inspiring for the neural architecture of autonomous humanoid robots, especially in enabling them to detect and respond quickly to danger thus to maintain their sustainability.
